# Integrative Analysis of Whole Exome Sequencing and Gene Expression to Uncover Genetic Mechanisms in Inflammatory Bowel Disease

**DOI:** 10.3390/genes17101306

**Published:** 2026-10-20

**Authors:** Babajan Banaganapalli, Waad Fahad Alharbi, Wejdan Abdullah Alzahrani, Ashraf A. El-Harouni, Mahmoud Almutadares, Khalidah Khalid Nasser, Omar I. Saadah, Mahmoud Mosli, Ragdah Hussain Arif, Hadeel A. Alsufyani, Dareen Alyousfi, Noor Ahmad Shaik

**Affiliations:** 1 Department of Genetic Medicine, Faculty of Medicine, King Abdulaziz University, Jeddah 21589, Saudi Arabia; 2 Princess Al-Jawhara Al-Ibrahim Center of Excellence in Research of Hereditary Disorders, King Abdulaziz University, Jeddah 21589, Saudi Arabia; 3 Department of Medical Laboratory Sciences, Faculty of Applied Medical Sciences, King Abdulaziz University, Jeddah 21589, Saudi Arabia; 4 Department of Pediatrics, Faculty of Medicine, King Abdulaziz University, Jeddah 21589, Saudi Arabia; 5 Department of Internal Medicine, Faculty of Medicine, King Abdulaziz University, Jeddah 21589, Saudi Arabia; 6 Department of Medicine, King Abdulaziz University, Jeddah 21589, Saudi Arabia; 7 Department of Medical Physiology, Faculty of Medicine, King Abdulaziz University, Jeddah 21589, Saudi Arabia; 8 Department of Clinical Biochemistry, Faculty of Medicine, King Abdulaziz University, Jeddah 21589, Saudi Arabia

**Keywords:** immune-related genes, rare variants, protein–protein interaction network, candidate genes, functional enrichment

## Abstract

**Background/Objectives**: Inflammatory bowel disease (IBD) is a chronic immune-mediated disorder of the gastrointestinal tract. This study aimed to nominate candidate genes and pathways for IBD by integrating exome sequencing and gene expression data. **Methods**: Whole-exome sequencing (WES) was performed on 10 patients with Crohn’s disease (CD). Differential expression was analyzed with GEO2R in an independent RNA-seq dataset (GSE193677; 2490 biopsies from 1162 subjects). Immune-related differentially expressed genes (DEGs) were identified with ImmPort, hub genes by protein-protein interaction analysis, and enriched pathways with Enrichr. Missense variants were modeled with AlphaFold 3. **Results**: WES identified 484 genes with rare coding variants in all 10 patients, and 732 genes were differentially expressed in CD or ulcerative colitis, 234 of them immune-related. Network, pathway, and database filtering prioritized *ATM*, *CSF1R*, *FN1*, and *IL11*, which together carry eight rare missense variants, with in silico predictions ranging from tolerated to deleterious. Enriched pathways included cytokine and interleukin signaling, and structural modeling predicted conformational changes. **Conclusions**: *ATM*, *CSF1R*, *FN1*, and *IL11* are candidate genes in immune-related pathways in IBD. Because the genomic and expression data came from different cohorts, these genes need replication and experimental validation.

## 1. Introduction

Inflammatory bowel disease (IBD) is a group of chronic, debilitating conditions characterized by progressive digestive tract inflammation, comprising two principal types: Ulcerative Colitis (UC) and Crohn’s disease (CD) [1]. The clinical manifestations of both types include persistent diarrhea, abdominal pain, bloody stool, fatigue, loss of appetite and weight loss. UC is restricted to the colon and rectum, presenting as continuous non-transmural inflammation of the mucosal layer that typically originates in the rectum and extends upward [2]. Conversely, CD can affect any segment of the gastrointestinal tract presenting as discontinuous patches (skip lesions) with transmural inflammation, penetrating through all layers of the intestinal wall [3]. Disease course and severity vary considerably depending on the location and extent of inflammation [4]. Beyond individual health impacts, IBD imposes substantial societal burdens through reduced educational attainment, workplace absenteeism, and healthcare costs [5].

According to the Global Burden of Disease (GBD) study, the number of people with IBD worldwide increased by 83.8% from 1990 to 2017, reaching 6.8 million [6]. Later GBD rounds revised the input data and models and gave lower prevalence estimates, so figures from different rounds should not be compared directly. The GBD 2021 analysis estimated 3.83 million prevalent cases and 375,140 incident cases of IBD in 2021, increases of 76.5% and 88.3% since 1990, and projected continued declines in age-standardized rates up to 2050 [7]. Estimates built from population-based cohorts are higher. A 2025 analysis of 522 population-based studies covering 1920–2024 across 82 global regions showed that the rise of IBD follows predictable epidemiologic stages (emergence, acceleration in incidence, and compounding prevalence), and current estimates place the number of people living with IBD at more than 7 million worldwide [8,9].

Although the exact etiology of IBD remains unknown, current evidence suggests that the disease arises from a dysregulated immune response in genetically susceptible individuals triggered by environmental factors including microbial and luminal antigens [10]. Furthermore, multiple pathogenic factors have been implicated in UC and CD including altered environmental exposures, genetic susceptibility variants, quantitative and qualitative alterations in gut microbiota composition, and aberrant immune responses [1]. Notably, genome-wide association studies (GWAS) have identified key susceptibility loci such as *IL23R*, *NOD2*, and *ATG16L1*, implicating the IL-23/Th17 axis, innate immune recognition, and autophagy pathways in disease pathogenesis [11].

Despite these insights and the identification of potentially relevant environmental, genetic, microbial, and immunological variables, a comprehensive understanding of the pathophysiology of IBD remains elusive [12]. Furthermore, the heterogeneous nature and the complexity of IBD, combined with incomplete knowledge of contributing genetic factors, clinical phenotype variability and drug pharmacokinetics, continue to limit disease understanding and therapeutic options [13]. While GWAS have successfully identified susceptibility loci, these studies primarily detect common variants with modest effect sizes and do not capture the functional consequences of genetic variation. Integrating transcriptomic data with exome sequencing can help to narrow this gap by asking whether genes that carry rare coding variants also belong to the immune networks that are dysregulated in inflamed intestinal tissue.

We hypothesized that combining whole-exome sequencing with transcriptomic analysis would nominate immune-related genes that carry rare coding variants in IBD patients and interact with immune genes that are dysregulated in IBD tissue, providing candidates for future functional and therapeutic studies.

This study aims to explore the genetic pathways underlying IBD pathogenesis through the integration of gene expression profiling and exome sequencing data. The specific objectives are to: (1) identify differentially expressed genes (DEGs) between IBD patients and healthy controls; (2) detect exonic variants in IBD-associated genes; (3) link variant-carrying genes to immune-related DEGs through protein–protein interaction network analysis; (4) characterize dysregulated molecular pathways implicated in IBD pathogenesis; and (5) identify candidate genes that could be tested as biomarkers or therapeutic targets in future experimental and clinical studies.

## 2. Materials and Methods

**Figure 1:**
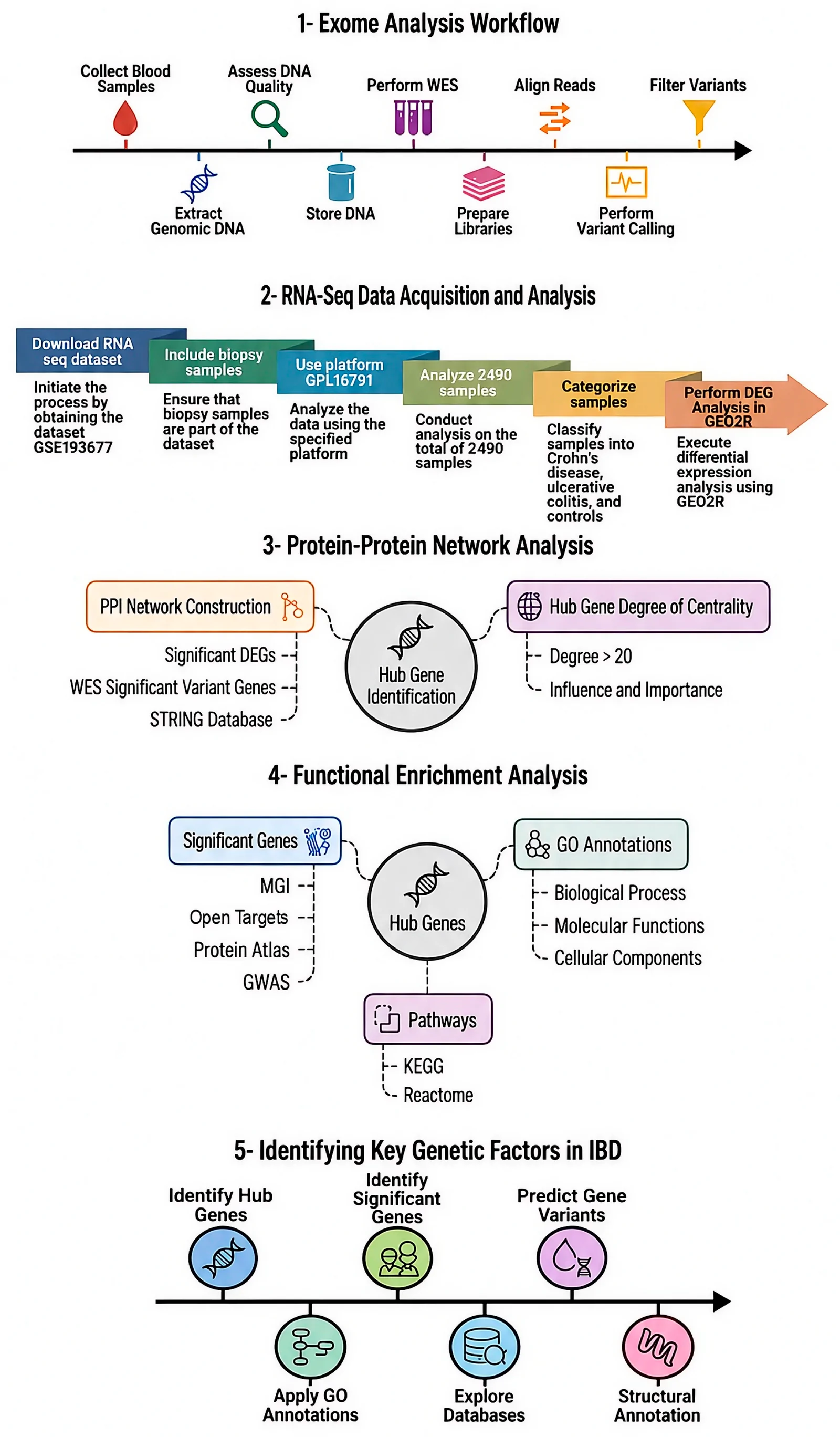
Integrated workflow for identifying key genetic factors in inflammatory bowel disease. The analysis pipeline combines (1) exome sequencing for variant discovery, (2) RNA-seq differential expression analysis (GSE193677), (3) protein-protein interaction network construction to identify hub genes, (4) functional enrichment analysis using GO annotations and pathway databases, and (5) integration of genomic, transcriptomic, and network data to prioritize candidate genes for IBD.

The study combined whole-exome sequencing (WES) of 10 IBD patients with differential gene expression (DEG) analysis of an independent public RNA-seq cohort (GSE193677). Because the two data sets come from different individuals, they were integrated at the gene level, not at the level of individual patients. The integration followed five steps, which correspond to the five panels of Figure 1. First, genes carrying rare coding variants in all 10 exomes were defined as the WES gene set (Section 2.1). Second, immune-related DEGs were defined from GSE193677 and ImmPort (Section 2.2 and Section 2.3). Third, the two gene sets were merged and analyzed as a single protein–protein interaction (PPI) network, and hub genes were selected by degree centrality, so that WES genes and immune DEGs were connected through shared interaction partners (Section 2.4). Fourth, hub genes were tested for KEGG and Reactome pathway enrichment; hub genes from the WES gene set that were annotated to the enriched immune pathways were retained, because only genes carrying variants in our patients could be followed up at the variant level, and these genes were scored against eight evidence criteria drawn from MGI, the published literature, the Open Targets Platform, the Human Protein Atlas and the GWAS Catalog (Section 2.5 and Section 2.6). Fifth, the genes with the strongest prior links to immune function or IBD were searched for rare missense variants in the individual patient exomes, and these variants were annotated, characterized and modeled (Section 2.7, Section 2.8 and Section 2.9).

### 2.1. Whole Exome Analysis Methods

#### 2.1.1. Sample Collection and DNA Extraction

Genomic DNA was extracted from peripheral blood samples collected from the study participants using the QIAamp DNA blood kit (C# 51104) following the manufacturer’s protocol. To assess the quality of the DNA extraction, DNA concentration and purity were measured using a Nanodrop spectrophotometer (ND-1000 UV–VIS). The integrity of the DNA was evaluated by 1% agarose gel electrophoresis, and samples were stored at −20 °C until they were used for genetic analysis.

#### 2.1.2. Whole Exome Sequencing (WES) Data Processing

The study included 10 Saudi patients with Crohn’s disease (CD) recruited from King Abdulaziz University Hospital, Jeddah (6 males and 4 females; Table S1). Age at diagnosis ranged from 6 months to 29 years. Recorded disease features included esophageal involvement (erosions with hemorrhagic gastropathy, *n* = 1; severe stenosis, *n* = 1), ileal stricture with enterocolonic fistula (*n =* 1), pan-ileocolonic ulceration (*n* = 1), moderate to severe colitis (*n* = 1), bloody diarrhea with vomiting (*n* = 1) and failure to thrive (*n* = 2); clinical features were not recorded for two patients. Extraintestinal and associated features included skin lesions (*n* = 2), osteopenia (*n* = 1), and hyperlipidemia (*n* = 1). Two patients had undergone colectomy, and five had received anti-TNF therapy (infliximab, *n* = 4; adalimumab, *n* = 1). All patients were unrelated. Whole exome sequencing (WES) was performed on DNA from these 10 patients by Macrogen Inc. (Seoul, Republic of Korea). DNA quantity and quality were checked by PicoGreen assay and agarose gel electrophoresis. Genomic DNA (average concentration 60 ng/μL) was sheared to a target peak size of 150–200 bp with a Covaris LE220 focused-ultrasonicator (Covaris, Woburn, MA, USA), followed by end repair, A-tailing, ligation of Agilent adapters and PCR amplification of the adapter-ligated fragments, according to the Agilent SureSelectXT library preparation protocol. Library size and quality were checked on a TapeStation D1000 ScreenTape (Agilent Technologies, Santa Clara, CA, USA). After capture, libraries were quantified by qPCR (KAPA Library Quantification Kit; Kapa Biosystems, Inc., Wilmington, MA, USA) and sequenced as 2 × 151 bp paired-end reads on an Illumina NovaSeq 6000 (Illumina Inc., San Diego, CA, USA). 

The Agilent SureSelect exome capture kit V7.0 was employed to target all exonic regions of protein-coding genes listed in the Consensus coding sequence (CCDS) and RefSeq databases. Ultra-long 120-mer biotinylated cRNA library baits were used for hybridization with the fragmented DNA. The capture target used for coverage calculations spans approximately 49.7 Mb.

#### 2.1.3. Data Acquisition of Whole Exome Sequencing Analysis

Read quality was assessed with FastQC (version 0.12.1). Raw paired-end reads (FASTQ) were aligned to the GRCh38 human reference genome with the BWA-MEM algorithm of BWA (version 0.7.19). Each sample was then processed individually following the GATK Best Practices workflow with GATK (version 4.6.1.0): duplicate reads were marked, base quality scores were recalibrated using known variant sites, and variants were called per sample with HaplotypeCaller. SAMtools (version 1.21) was used for alignment processing and summary statistics. According to the provider’s quality control report, sequencing produced a mean of 63.6 million reads per sample (range 53.0–78.0 million), with 93.7% of bases at Q30 or above. The mean on-target depth was 91.4× (range 71.9–114.4×), and on average 97.0% and 91.2% of target bases were covered at ≥10× and ≥20×, respectively (Table S2). Variants with a Phred-scaled quality score >30 and a read depth of at least 30 were retained. Variants were then filtered in three steps: intronic, other noncoding and synonymous variants were removed; only variants in protein-coding transcripts were kept; and variants with a minor allele frequency (MAF) >0.01 in the 1000 Genomes Project phase 3 or in gnomAD v2.1 exomes or genomes were removed. These criteria follow the usual design of exome studies that search for rare variants with larger effects on protein function. Nonsynonymous coding variants are the class most likely to change protein structure, and they can be assessed with protein-level tools such as SIFT, PolyPhen-2 and structural modeling. Intronic and synonymous variants are poorly captured by exome enrichment, which covers only short flanking intronic sequence, and their effects cannot be interpreted reliably from exome data alone. The MAF threshold of 0.01 limits the analysis to rare variants. Common IBD risk alleles of small effect have already been mapped by GWAS [11], and our aim was to detect rarer variants that GWAS arrays do not capture well. As a consequence, noncoding regulatory variants, most splice-region variants and common variants were not evaluated. For each patient, genes with at least one variant passing all filters were listed, and genes present in all 10 patients (the same gene, not necessarily the same variant) were identified with the Venny 2.1.0 tool (https://bioinfogp.cnb.csic.es/tools/venny/; accessed on 15 July 2025).

### 2.2. Data Acquisition of Gene Expression Analysis

We collected the Gene expression Dataset from the GEO database. We downloaded the IBD RNA-seq dataset GSE193677 from (https://www.ncbi.nlm.nih.gov/geo/; accessed on 1 August 2025). The dataset includes 2490 intestinal biopsy samples (712 inflamed and 1778 non-inflamed) from 1162 subjects (498 Crohn’s disease, 421 ulcerative colitis, and 243 controls) [14]. Biopsies were taken from several intestinal regions, and many subjects contributed more than one biopsy. The Platform GPL16791 (Illumina HiSeq 2500) was used.

### 2.3. Differential Gene Expression Analysis and Immune-Related Genes

Differentially expressed genes (DEGs) between IBD patients and healthy controls were identified with GEO2R (https://www.ncbi.nlm.nih.gov/geo/geo2r/; accessed on 15 August 2025), which analyzes RNA-seq count data with DESeq2. Samples were first grouped as CD, UC or healthy control, and two pairwise comparisons were performed: CD versus healthy controls and UC versus healthy controls. Biopsies were grouped by diagnosis only, so inflamed and non-inflamed biopsies, and biopsies from all intestinal regions, were pooled within each group. GEO2R reports the log2 fold change of the control group relative to the disease group; we reversed the sign so that positive values indicate higher expression in IBD. *p* values were adjusted with the Benjamini–Hochberg false discovery rate method, and genes with an adjusted *p* value < 0.05 and an absolute log2 fold change ≥ 0.75 were considered differentially expressed. Genes meeting these criteria in either comparison were combined into a single, non-redundant list of IBD DEGs. GEO2R treats every biopsy as an independent sample and cannot include subject, biopsy location or inflammation status as covariates. Many subjects contributed several biopsies, and disease subtype, inflammation and biopsy site all influence intestinal gene expression, so the *p* values from this analysis are likely to be optimistic and part of the DEG signal may reflect inflammation and sampling site, not disease status alone. We therefore treat the DEG list as a screening step. To find immune-related genes, we downloaded 2483 immune genes from ImmPort (https://www.immport.org/resources; accessed on 18 August 2025). Immune-related DEGs were defined as the overlap between the significant DEGs and the ImmPort list, identified with Venny 2.1.0 (https://bioinfogp.cnb.csic.es/tools/venny/; accessed on 18 August 2025).

### 2.4. Selection of the Hub Genes: Protein-Protein Interaction Network Analysis

To identify the Hub genes, we used Cytoscape (version 3.10.3; https://cytoscape.org) with StringApp (version 2.1.1), which queries the STRING database (version 12.0), with the default interaction confidence score cutoff of 0.4, to determine protein-protein interaction network analysis. Immune-related differentially expressed genes, and WES genes were combined and used as input to determine the protein-protein interaction. A degree of centrality of >20 was used as a criterion to select the significant genes.

### 2.5. Functional Enrichment Analysis (KEGG and Reactome)

To identify the common immune pathways related to the shared genes between WES and gene expression analysis, we performed enrichment analysis using Enrichr (https://maayanlab.cloud/Enrichr/; accessed on 24 August 2025) to identify the role of these genes in KEGG and Reactome pathways. Significant genes from protein-protein interaction (Hub genes) were used as input to identify the genes enriched in KEGG and Reactome Pathways.

Pathways with a Benjamini–Hochberg adjusted *p* < 0.05 were considered significantly enriched. Enrichr computes enrichment with Fisher’s exact test against the genes annotated in each pathway library. We did not supply a custom background, so the reference set was Enrichr’s default gene universe for the KEGG 2025 and Reactome Pathways 2024 libraries. We also used the ClueGO plugin (version 2.5.10; https://apps.cytoscape.org/apps/cluego; accessed on 24 August 2025) in Cytoscape to visualize the pathway networks, with the whole-genome *Homo sapiens* annotation as the reference set. In ClueGO, enrichment was assessed with a two-sided hypergeometric test and Bonferroni step-down correction, and related terms were merged by GO term fusion. The ClueGO category network was built from term overlap at *p* < 0.05. The input genes had already been selected for immune function and network centrality, so a genome-wide background will favor immune pathways. The enrichment results therefore describe which pathways are represented among the hub genes and are not independent evidence that these pathways are disrupted in IBD.

### 2.6. Identification of Significant Genes (Gene Annotation)

We performed a set of functional biological analyses on the 16 genes using several platforms to identify the role of these genes in the pathogenesis of IBD. We utilized these tools to identify the phenotype characterization, and expression of the genes in the cells and their role in the pathogenesis of IBD. We used the Mouse Genome Informatics (MGI) database (https://www.informatics.jax.org; accessed on 30 August 2025) to identify the genes to disease correlation in both humans and mice, in addition to the phenotypes. Furthermore, protein atlas (https://www.proteinatlas.org; accessed on 30 August 2025) was employed to identify the expression of these genes in specific tissues, particularly in the colon and immune systems. GWAS (genome-wide association studies) catalog (https://www.ebi.ac.uk/gwas/ and https://www.informatics.jax.org; accessed on 10 September 2025) was used to identify the common genomic loci associated with IBD phenotypes. Open Target platform (https://platform.opentargets.org) was used to identify gene targeted drugs. Each of the 16 genes was scored as positive (1) or negative (0) for eight criteria: (1) annotation to an enriched immune pathway (KEGG or Reactome); (2) an immune or digestive system phenotype in MGI mouse models; (3) at least one published study linking the gene to IBD, identified by a PubMed search; (4) association with CD or UC in the Open Targets Platform (release 25.09; accessed on 25 September 2025); (5) a drug association in the Open Targets Platform; (6) detection in blood immune cells in the Human Protein Atlas (version 24); (7) any association in the GWAS Catalog (accessed on 25 September 2025); and (8) being an HLA gene.

### 2.7. Candidate Gene Prioritization

For the eight genes selected by functional annotation, all variants that passed the filters in Section 2.1.3 were retrieved from the individual patient exomes, and missense variants were taken forward for pathogenicity prediction. Variant annotation was performed with the Ensembl Variant Effect Predictor (VEP; version 113) (https://asia.ensembl.org/info/docs/tools/vep/index.html; accessed on 30 September 2025). Population allele frequencies of the retained variants, overall and in the Middle Eastern population, were taken from gnomAD v4.1 exomes through the Ensembl VEP REST service, because gnomAD v2.1 has no Middle Eastern population. Variants were classified with SIFT (version 6.2.1; deleterious when ≤0.05) and PolyPhen-2 (version 2.2.3; possibly damaging 0.446–0.908; probably damaging >0.908; HumVar model as reported by VEP). Only genes carrying at least one rare missense variant were retained as final candidates, because the later steps (SIFT, PolyPhen-2, AlphaFold modeling and DUET stability prediction) are designed to assess single amino acid substitutions. We did not apply ACMG/AMP classification, and we refer to the retained variants as candidate variants, not pathogenic variants.

### 2.8. Integrative Functional Characterization and Druggability Assessment

Candidate genes were evaluated using an integrative functional annotation approach. Drug–gene interactions were retrieved from DrugBank (version 5.1.13; https://go.drugbank.com) and the Drug Gene Interaction Database (DGIdb version 5.0; https://www.dgidb.org) to assess druggability. Protein domain mapping was performed using UniProt (release 2025_03; https://www.uniprot.org; accessed on 5 October 2025) and Pfam annotations accessed through InterPro (https://www.ebi.ac.uk/interpro/; accessed on 5 October 2025) to localize variants within functionally important regions. Tissue expression was obtained from the Genotype-Tissue Expression project (GTEx release v8; median transcripts per million [TPM] per tissue; https://gtexportal.org; accessed on 5 October 2025), and expression in 18 sorted blood immune cell types from the Human Protein Atlas blood atlas (version 24; normalized TPM [nTPM]; https://www.proteinatlas.org; accessed on 5 October 2025); both were accessed in October 2026. Pathway involvement was assessed using Kyoto Encyclopedia of Genes and Genomes (KEGG release 115; https://www.genome.jp/kegg; accessed on 5 October 2025) annotations to identify convergence on inflammatory signaling pathways. The four genes were then ranked for potential druggability using the combined evidence of drug–gene interactions, variant location in functional domains, disease-relevant expression, pathway membership and published preclinical studies. DGIdb aggregates drug–gene interaction claims from curated sources, including DrugBank and ChEMBL, and DrugBank records drug targets reported in the literature and in regulatory documents [15,16]. These interactions therefore rest on published experimental or clinical evidence that a compound binds or modulates the protein, mostly for indications other than IBD. They were not validated in IBD and were not tested experimentally in this study; we used them only as an indicator of pharmacological tractability.

### 2.9. Protein Modeling and Functional Analysis

Missense variants were further analyzed by structural modeling. Structural modeling was performed for both wild-type and mutant proteins using AlphaFold 3 (AlphaFold Server, https://alphafoldserver.com/; accessed on 10 October 2025). Variants were analyzed for structural deviations using PyMOL molecular visualization tools (version 3.1) (https://www.pymol.org; accessed on 10 October 2025). Furthermore, changes in protein stability were predicted using the DUET web server (https://biosig.lab.uq.edu.au/duet/; accessed on 10 October 2025), with a focus on variants that could alter protein folding or function. The functional implications of these structural changes were evaluated in the context of pathways relevant to IBD.

## 3. Results

### 3.1. DNA Quality and Whole Exome Sequencing

#### 3.1.1. DNA Extraction and Quality Assessment

High-quality genomic DNA was effectively isolated from the peripheral blood samples of all participants in the study using the QIAamp DNA blood kit (C# 51104). A Nanodrop spectrophotometer was employed to verify DNA concentration and purity, while 1% agarose gel electrophoresis was used to confirm DNA integrity.

#### 3.1.2. Sequencing Metrics and Read Alignment

Whole exome sequencing (WES) was conducted on DNA samples from 10 affected individuals. Reads were mapped to the GRCh38 human reference genome with a mean on-target depth of 91.4× (Section 2.1.3; Table S2), and all retained variants had a Phred quality score above 30. The filtering process eliminated noncoding, synonymous, and common variants (minor allele frequency > 0.01), resulting in a dataset of high-confidence variants.

### 3.2. Whole Exome Sequencing Analysis

The whole exome sequencing provided a total number of 972,982 of variants among the 10 patients. These variants were further filtered out by excluding synonymous variants and intronic variants from which resulted in total of 387,522 variants. Also, the number of variants were narrowed down by filtering out the variants based on the transcript biotype to select the protein coding variants only which provided 309,083 total number of variants. Three filtration criteria were additionally applied using Minor Allelic Frequency (MAF) to only select the variants with less than or equal to (0.01) in three databases (1000 Genomes Project phase 3, gnomAD v2.1 exomes and gnomAD v2.1 genomes), narrowing it down to 61,268 variants. Further, the passing variants were 47,968. Table 1 illustrate Total Number of Variants Before and After Filtration.

### 3.3. Identification of the Shared Genes in WES Analysis

Summed over the 10 patients, 24,555 genes carried at least one filtered variant (Table 1); a gene found in several patients is counted once for each patient. To identify the common genes among the patients, the list of genes of all 10 patients were intersected and 484 genes carried at least one rare coding variant in every one of the 10 IBD patients (the same gene, but not necessarily the same variant).

### 3.4. Identification of Significantly Differentially Expressed Immune-Related Genes

**Figure 2:**
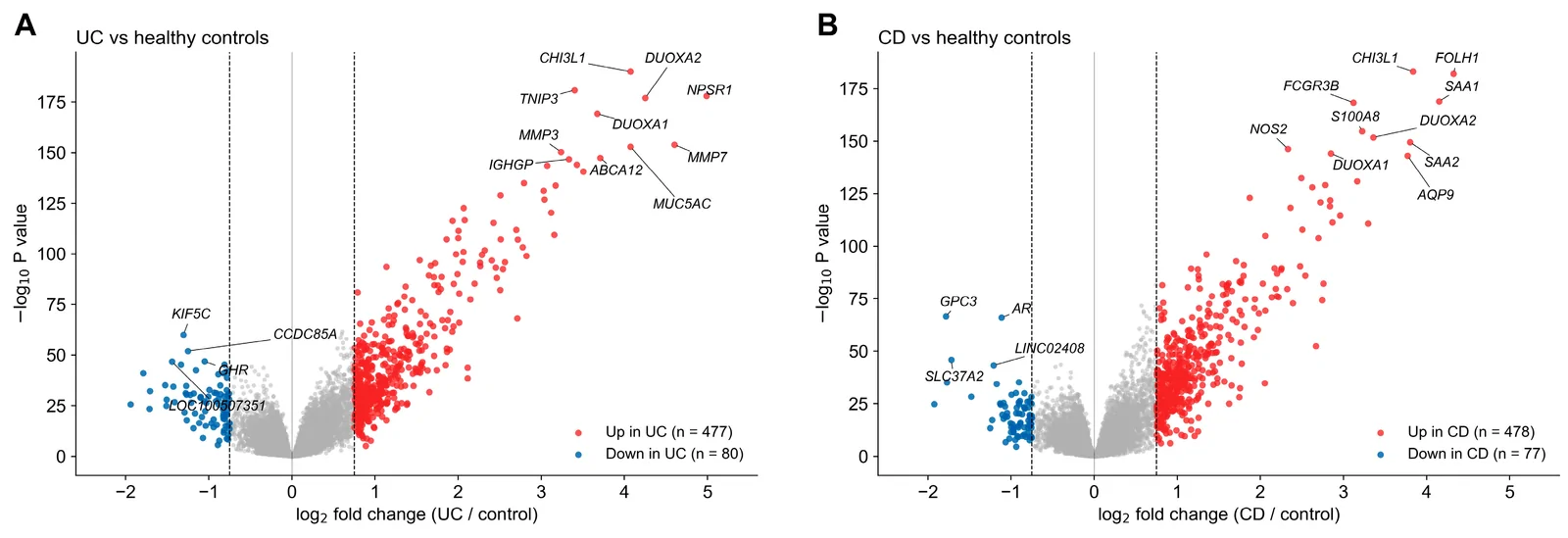
Volcano plots of differential expression in GSE193677 for (**A**) UC versus healthy controls and (**B**) CD versus healthy controls. Positive log2 fold change indicates higher expression in the disease group. Red and blue points are genes up- and down-regulated in disease (Benjamini–Hochberg adjusted *p* < 0.05 and |log2 fold change| ≥ 0.75); dashed lines mark the fold-change threshold. The 10 most significant up-regulated and 4 most significant down-regulated genes are labeled.

To investigate the role of the immune-related genes related to IBD of both types, UC and CD, we downloaded an RNA-seq from GEO (https://www.ncbi.nlm.nih.gov/geo/; accessed on 1 August 2025) to perform a differential expression analysis among the IBD intestinal samples. 557 genes were significantly differentially expressed in UC versus controls and 555 in CD versus controls (Figure 2A,B). Because 380 genes were significant in both comparisons, the combined list contained 732 unique DEGs: 622 up-regulated and 109 down-regulated in IBD relative to controls, and one that changed direction between the two comparisons. Furthermore, to identify the immune-related genes, we identified the shared genes among the 732 DEGs and 2483 immune-related genes from ImmPort, and 234 differentially expressed immune genes were identified. Fold changes in the two comparisons were closely correlated (r = 0.88 across 21,422 genes), and 379 of the 380 shared DEGs changed in the same direction. Of the 234 immune-related DEGs, 208 were significant in the CD comparison; the other 26 were also altered in CD (adjusted *p* < 0.05, same direction) but fell below the fold-change threshold. We combined the two comparisons because CD and UC share most of their genetic risk loci and inflammatory pathways [17], and in these data the immune DEG signal came mainly from CD.

### 3.5. Identification of Hub Genes from Whole Exome Analysis and Gene Expression Analysis

**Figure 3:**
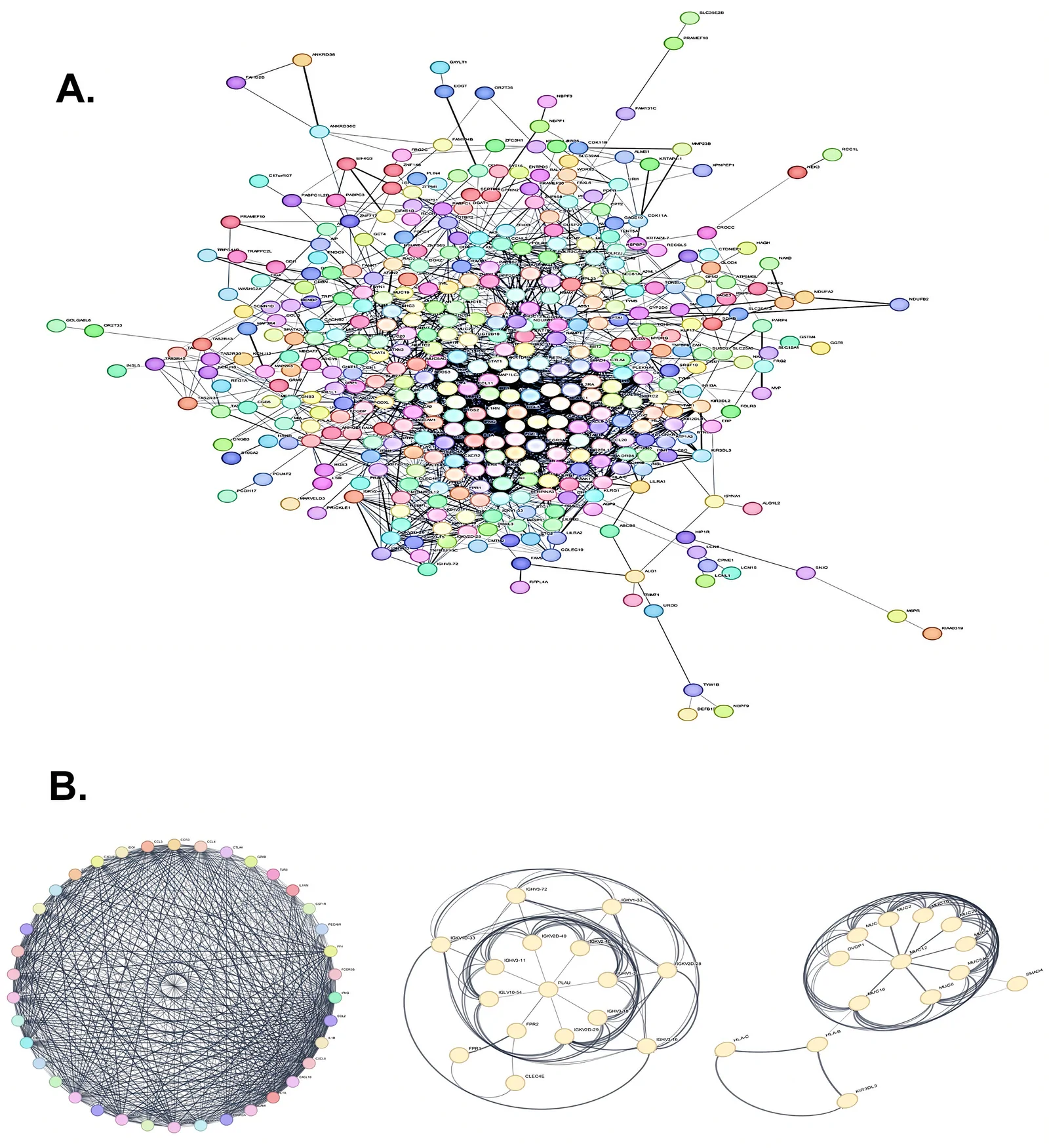
Protein–protein interaction network analysis of the hub genes. (**A**) Protein–protein interaction network of the combined immune-related differentially expressed genes and whole-exome sequencing genes. (**B**) Sub-networks of the hub genes (degree of centrality > 20).

The immune related DEGs along with genes determined from the WES analysis were combined, giving 716 unique genes (MUC5AC and LILRB3 were present in both sets). To find the hub genes that are shared among the immune-related differentially expressed genes and WES genes, the Cytoscape tool (https://cytoscape.org) using StringApp was implemented to identify and analyze the protein-protein interactions, and 72 Hub genes exhibited degree of centrality values exceeding 20. Of the 72 hub genes, 17 came from the WES gene set, 54 from the immune-related DEGs, and one (H3C12) was a network interactor present in neither list. Figure 3 displays the protein-protein interaction network of the hub genes.

### 3.6. Functional Enrichment Analysis

**Figure 4:**
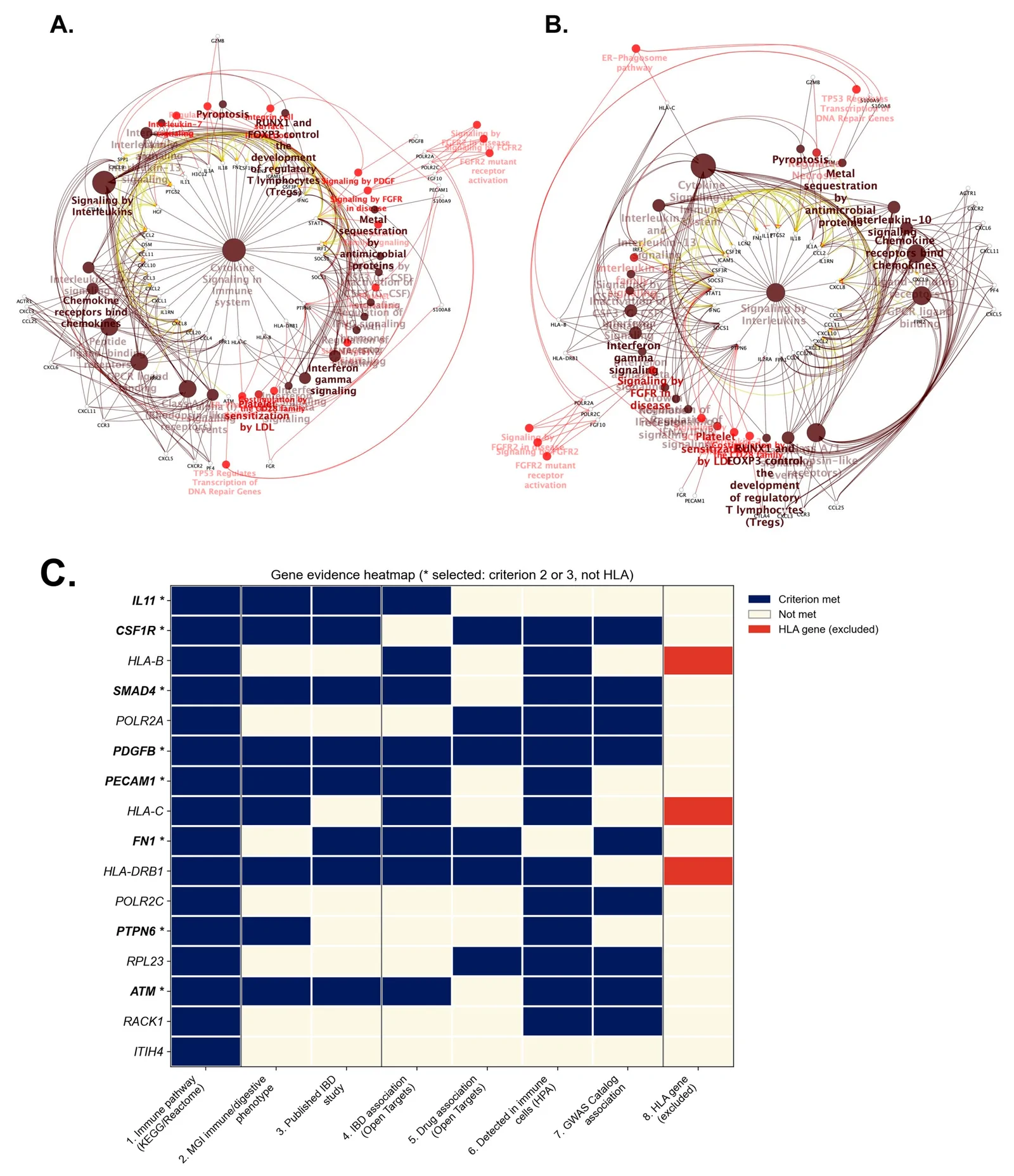
Pathway Enrichment and Gene Association Profiles. (**A**) Functional network of enriched KEGG pathways showing crosstalk between immune signaling and metabolic modules. (**B**) Reactome pathway network highlighting granular molecular interactions, including pyroptosis and regulatory T-cell development. For both networks, nodes represent pathways and edges indicate gene overlap. (**C**) Evidence heatmap for the 16 hub genes annotated to the enriched immune pathways. Dark blue squares indicate that the criterion was met and light squares that it was not; red squares mark HLA genes, which were excluded. * Genes were selected if they met criterion 2 or 3 and were not HLA genes; the eight selected genes are shown in bold and marked with an asterisk. Abbreviations: MGI, Mouse Genome Informatics; HPA, Human Protein Atlas; GWAS, genome-wide association study.

To identify the common pathways among the genes, we used Enrichr platform (https://maayanlab.cloud/Enrichr/; accessed on 24 August 2025) where the 72 hub genes were inserted as input to identify the common KEGG and Reactome pathways. Among the common pathways is signaling by Interleukins, and cytokine signaling in the immune system, which suggests the role of these genes in the immune system. Figure 4A,B displays the common reactome and KEGG pathways. Of the hub genes annotated to these enriched KEGG and Reactome immune pathways, the 16 that belonged to the WES gene set were taken forward, because only genes carrying variants in our patients could be followed up at the variant level: *IL11*, *CSF1R*, *HLA-B*, *SMAD4*, *POLR2A*, *PDGFB*, *PECAM1*, *HLA-C*, *FN1*, *HLA-DRB1*, *POLR2C*, *PTPN6*, *RPL23*, *ATM*, *RACK1*, and *ITIH4*.

### 3.7. Functional Annotation Analysis

Using biological functional analysis methods, different tools were employed to identify the significant genes associated with IBD, including phenotypes, expression, and GWAS. Eight of the 16 genes met the selection criteria described in Section 2.6 (Figure 4C): *IL11*, *CSF1R*, *SMAD4*, *PDGFB*, *PECAM1*, *FN1*, *ATM* and *PTPN6*. Seven of them had an immune or digestive phenotype in MGI mouse models, and all except *PTPN6* had at least one published study linking them to IBD; *FN1* qualified through published evidence alone. *HLA-C* and *HLA-DRB1* also met these criteria but were excluded as HLA genes (HLA excluded because the high polymorphism of the HLA region makes rare-variant calls from short-read exome data unreliable), and *HLA-B*, *POLR2A*, *POLR2C*, *RPL23*, *RACK1* and *ITIH4* met neither criterion.

### 3.8. Candidate Gene Prioritization

The most biologically relevant transcript was chosen for each of the eight candidate genes from the WES results. The Ensembl Variant Effect Predictor (VEP) was used to annotate variants, with emphasis on missense variants predicted to have functional impacts. Variants were classified as either deleterious or benign using predictive tools such as PolyPhen-2 and SIFT, with choosing only missense variants. This process further refined the candidate list to four genes: *ATM*, *CSF1R*, *FN1*, and *IL11*. *SMAD4*, *PDGFB*, *PECAM1* and *PTPN6* were not retained because none of them carried a rare missense variant that passed the filters. All four genes came from the WES gene set shared by the 10 patients, and none of them met the differential expression thresholds in GSE193677. Their link to the expression data is therefore through their interaction partners among the immune-related DEGs in the PPI network (Figure 3); the variant and expression data were not measured in the same individuals.

### 3.9. Finalized Variant Analysis

Eight rare missense variants were retained in the four genes (Table S3): *CSF1R* p.Ser930Cys and p.Ala123Gly; *ATM* p.Phe582Leu; *FN1* p.Thr2202Ala, p.Asp1450Glu, p.Arg1040Gly and p.Gly313Val; and *IL11* p.Met144Thr. All eight were homozygous. Because gnomAD v2.1, which was used for filtering, has no Middle Eastern population, the eight variants were also looked up in gnomAD v4.1. In gnomAD v4.1 all eight are rare overall (allele frequency ≤0.08%), but ATM p.Phe582Leu has a frequency of 2.3% in Middle Eastern exomes, and FN1 p.Asp1450Glu is absent from gnomAD (Table S3). The in silico predictions varied. *CSF1R* p.Ser930Cys was deleterious by SIFT (0.02) and possibly damaging by PolyPhen-2 (0.544). *CSF1R* p.Ala123Gly and *IL11* p.Met144Thr were deleterious by SIFT but benign by PolyPhen-2. *FN1* p.Asp1450Glu, p.Arg1040Gly and p.Gly313Val were deleterious by SIFT, with no PolyPhen-2 score available, and *ATM* p.Phe582Leu and *FN1* p.Thr2202Ala were tolerated by SIFT with low confidence.

### 3.10. Integrative Functional Characterization and Druggability Assessment

**Figure 5:**
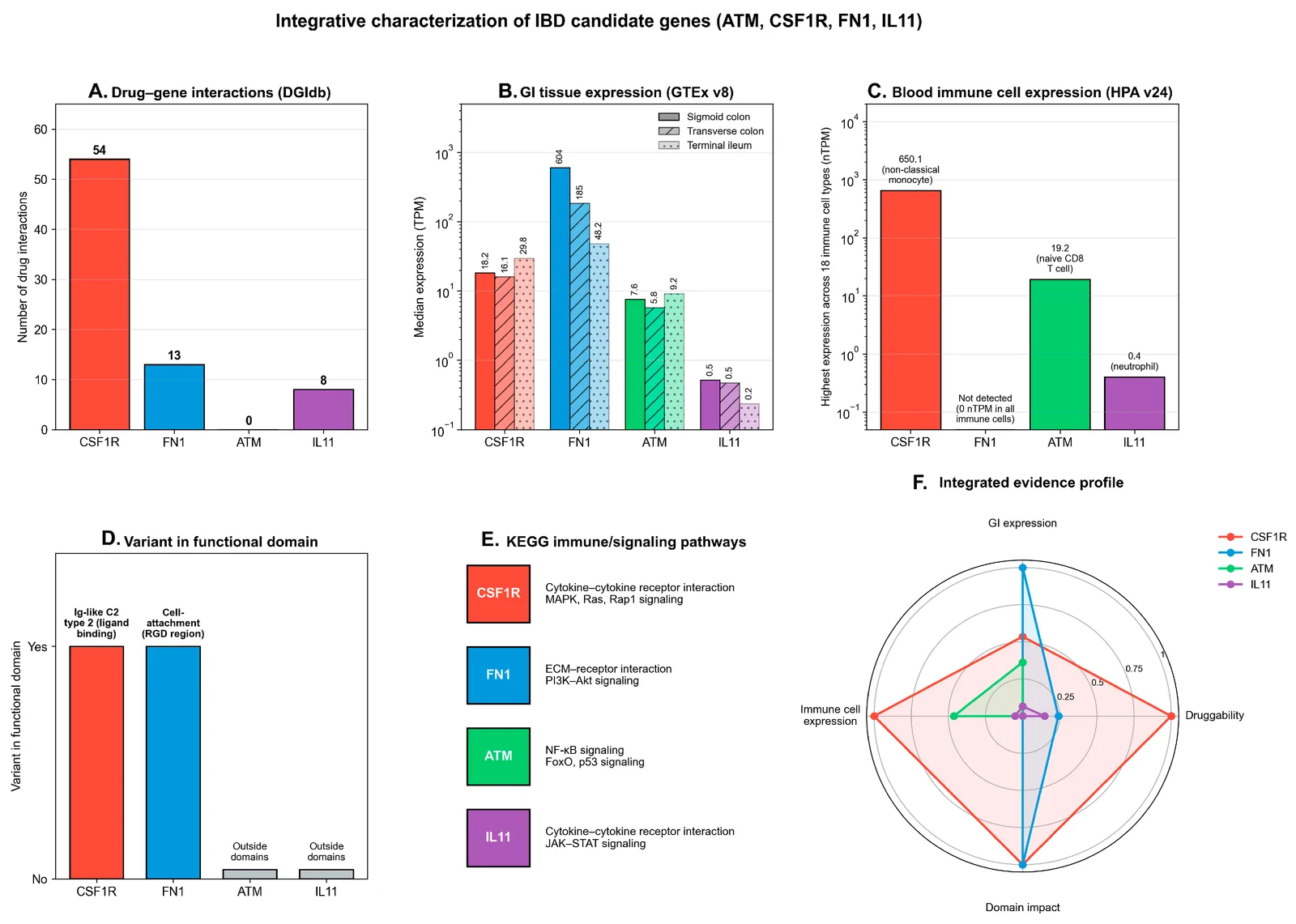
Integrative characterization of IBD candidate genes. (**A**) Number of drug–gene interactions from DGIdb. (**B**) Gastrointestinal tissue expression (GTEx v8, median TPM, log scale). (**C**) Expression across 18 blood immune cell types (Human Protein Atlas v24, nTPM, log scale). (**D**) Variant localization within functional protein domains. (**E**) KEGG pathway associations relevant to IBD pathogenesis. (**F**) Integrated evidence profile summarizing druggability, gastrointestinal and immune cell expression (log scale), and domain impact, each scaled from 0 to 1 across the four genes. Color coding: CSF1R (red), FN1 (blue), ATM (green), and IL11 (purple).

Integrative analysis of the four candidate genes as illustrated in Figure 5, ranked *CSF1R* as the most pharmacologically tractable candidate, with 54 documented drug interactions, including pexidartinib (an inhibitor approved by the FDA for tenosynovial giant cell tumor, not for IBD) and other CSF1R inhibitors, including cabiralizumab and emactuzumab, which have been tested in clinical trials, and the preclinical compound PLX5622. Protein domain mapping identified two variants in functionally critical regions: *CSF1R* p.Ala123Gly localized within the Ig-like C2-type 2 domain (amino acids 107–197), which constitutes part of the ligand-binding region essential for CSF-1 and IL-34 recognition, while *FN1* p.Asp1450Glu mapped to the cell-attachment domain (amino acids 1358–1631) containing the RGD motif critical for integrin binding and epithelial barrier function. In GTEx, *FN1* had the highest expression of the four genes in GI tissues, particularly in sigmoid colon (median 603.6 TPM) and transverse colon (185.3 TPM); *CSF1R* and *ATM* were expressed at lower levels (5.8–29.8 TPM), and *IL11* was barely detected (<1 TPM). Among blood immune cells in the Human Protein Atlas, *CSF1R* was highly expressed in monocytes (non-classical 650.1, intermediate 444.5 and classical 138.4 nTPM) and myeloid dendritic cells (139.1 nTPM), *ATM* was expressed at moderate levels across lymphocyte subsets (highest in naive CD8+ T cells, 19.2 nTPM), and *FN1* and *IL11* were essentially not expressed (≤0.4 nTPM). KEGG pathway analysis revealed convergence on inflammatory signaling cascades: *ATM* in NF-κB signaling, *CSF1R* in cytokine-cytokine receptor interaction and MAPK/Ras pathways, *FN1* in PI3K-Akt signaling, and *IL11* in JAK-STAT signaling. On these criteria *CSF1R* ranked first among the four candidates, based on its druggability, variant localization in a ligand-binding domain, elevated expression in disease-relevant immune cells, and published preclinical evidence that CSF1R inhibition reduces colitis severity in mice (discussed in Section 4). This ranking rests on computational and literature evidence and does not show that targeting *CSF1R* would benefit patients with IBD.

### 3.11. Structural Protein Modeling

**Figure 6:**
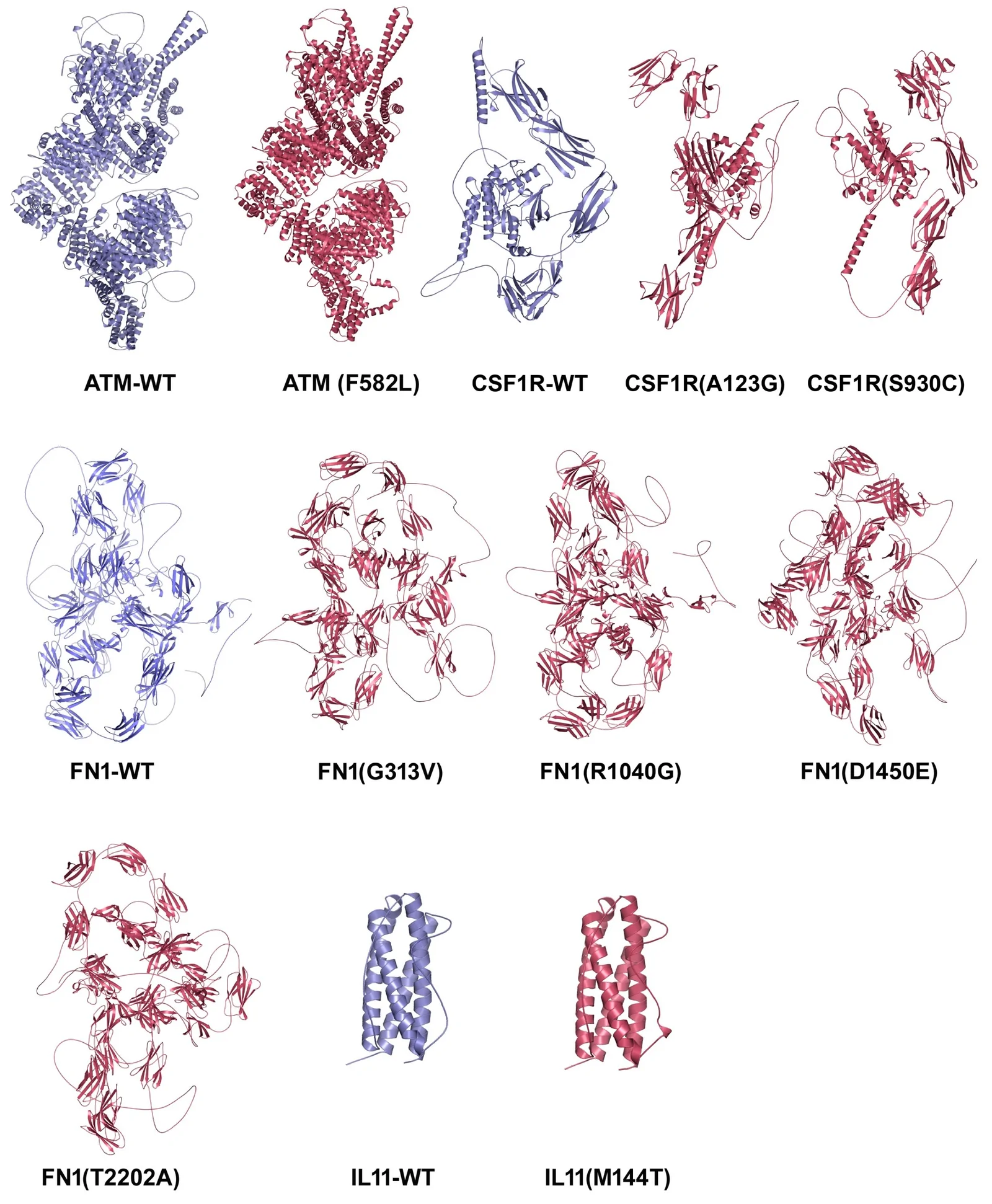
Structural protein modeling for the identified candidate gene variants comparing the structural models of wild type and mutant proteins and illustrating the conformational changes resulting from the specific amino acid substitutions.

Structural models of wild-type and mutant proteins showed conformational differences at the substituted residues. DUET predicted changes in protein stability, suggesting that the variants could affect protein folding and function. These are predictions from static models and do not show that the substitutions change protein activity; they indicate which variants are worth testing experimentally. Figure 6 compares the mutant and wild-type protein structures and the conformational changes associated with each amino acid substitution.

## 4. Discussion

Inflammation of the gastrointestinal system is the hallmark of the chronic, lifelong illness known as inflammatory bowel disease (IBD), which results from an interaction among environmental influences, immunological responses, and the host’s genetic susceptibility. Mucosal inflammation in CD can affect the tissue at full depth and contribute to problems, including strictures and fistulas. Conversely, mucosal inflammation in UC is limited to the colon [1]. However, in some instances, it is impossible to categorize IBD as either UC or CD [13]. Making a prompt and correct diagnosis, which calls for evaluating clinical history, laboratory results, imaging, endoscopy, and histopathology, continues to be one of the significant challenges associated with managing IBD [18]. In a research setting, determining the influence of the genetic factors, immune factors, and microbiota involved in the pathogenesis of chronic inflammation remains a challenge [10].

This study combined gene expression analysis and whole exome analysis to nominate candidate genes in IBD. The findings are associations derived from computational analyses. They point to genes and pathways that deserve further study, but they do not establish that these genes cause IBD or show how they contribute to it.

WES analysis provided a total of number of 972,982 variants among the 10 affected individuals. Multiple filtration criteria were applied which provided total of 47,968 variants among the 10 IBD patients. The criteria applied included exclusion of intronic and synonymous variants, so that nonsynonymous coding variants were retained. This was followed by selection of minor allele frequency of less than or equal to (0.01) in three databases (1000 Genomes Project phase 3, gnomAD v2.1 exomes and gnomAD v2.1 genomes) to identify the rare variants. Further, in the transcript biotype only protein coding was selected. Summed over the 10 patients, 24,555 genes carried at least one filtered variant. Additionally, a total 484 genes were common in all 10 patients.

To identify the significant genes related to the IBD progression and pathogenesis, transcriptomic analysis was conducted using RNA seq dataset to identify the differentially expressed genes among UC patients, CD patients and controls, using intestinal biopsies from several regions. The differential expression analysis identified 732 unique differentially expressed genes (557 in UC and 555 in CD compared with healthy controls). Furthermore, 234 of these genes were immune-related, suggesting a possible role in the pathogenesis of IBD, particularly the immune-related factors. Among these genes, 72 Hub genes were identified using protein-protein interaction network analysis on the genes identified from the Whole exome analysis and Immune differentially expressed genes.

Enrichment analysis identified 16 hub genes from the WES gene set that were annotated to the enriched KEGG and Reactome immune pathways. Figure 4A displays the common KEGG pathways which include cytokine-cytokine receptor interaction, chemokine signaling pathway, IL17 signaling pathways, and TNF Signaling pathway which all indicate the role of the identified genes in the immune related pathways.

Furthermore, Figure 4B shows the common Reactome pathways which included Chemokine receptors bind chemokines, signaling by interleukins, cytokine signaling in immune system and immune system pathways which emphasize the role of those genes in immune related pathways.

To identify the significant genes and further identify the contribution of the identified 16 genes in the disease process, functional biological analysis was utilized. Multiple tools and databases were employed to identify the functions of those genes in IBD pathogenesis. MGI databases was employed to identify detailed information about mouse knock out genes, phenotypes, and disease models. Protein atlas database was used to identify the expression level of the identified genes in immune cells and colon tissues. GWAS catalog was used to identify published genome-wide association studies, providing curated data on genetic variants associated with various diseases, including IBD. This database enables to identify validated disease-associated loci, cross-reference genetic findings, and understand the contribution of specific genetic variants to disease susceptibility across diverse populations. Additionally, open target platform databases provided information about gene targeted drugs.

Functional annotation narrowed the 16 genes to eight candidates. Genes were prioritized if they had an immune or digestive phenotype in mouse models or published evidence linking them to IBD, and HLA genes were excluded. Based on the functional annotation analysis, eight candidate genes were identified, which are *IL11*, *CSF1R*, *SMAD4*, *PDGFB*, *PECAM1*, *FN1*, *ATM*, and *PTPN6*.

Furthermore, these genes were analyzed in WES results for 10 IBD affected individuals. The most biologically relevant transcript was chosen for each of the eight candidate genes with emphasis on missense variants which are predicted to have functional impacts. This process led to identification of the final candidate list of genes which included several variants which are: *ATM*, *CSF1R*, *FN1*, and *IL11*.

Our findings align with a growing body of evidence from integrative genomic studies on autoimmune gastrointestinal disorders. A previous study, identified *LILRB1* and *PRSS3* variants in IBD families through similar WES and computational approaches, suggesting that innate immune dysfunction contributes to IBD pathogenesis [19]. Systems biology analyses of celiac disease have revealed shared molecular pathways with IBD, including IL-17 signaling, Th1/Th2 differentiation, and cytokine-cytokine receptor interactions [20,21,22]. These findings collectively support the existence of common molecular mechanisms underlying autoimmune enteropathies and support the use of integrative genomic approaches to nominate candidate genes for experimental follow-up.

### 4.1. CSF1R Gene

*CSF1R* gene encodes colony stimulating factor 1 receptor. The gene is located on chromosome 5 (5q32). It is a class III receptor tyrosine kinase that macrophages and microglia express on their cell surfaces. Microglia depend on CSF1R signaling to survive [23]. Functional annotation analysis indicated that this gene is associated with GI diseases such as colon adenocarcinoma and gastrointestinal stromal tumor and colorectal carcinoma. A study found that CSF-1 pathway activation contributes to Crohn’s disease, especially in anti-TNFα non-responders, and inhibiting CSF-1R reduced disease severity in mice, supporting trials in humans [24]. Importantly, Manthey et al. demonstrated that the selective CSF-1R inhibitor JNJ-40346527 (PRV-6527) significantly reduced inflammatory macrophage recruitment to the intestinal mucosa and suppressed murine T cell-mediated colitis [24].

WES analysis identified two rare missense variants in *CSF1R*. The first, p.Ser930Cys, was predicted to be deleterious by SIFT (0.02) and possibly damaging by PolyPhen-2 (0.544). The second, p.Ala123Gly, was predicted to be deleterious by SIFT (0) but benign by PolyPhen-2 (0.289). Structural protein modeling illustrated in Figure 6 indicated conformational changes in the protein structure due to the amino acid substitution. These preclinical findings, combined with our identification of potentially deleterious *CSF1R* variants in IBD patients and its high expression in monocytes and myeloid dendritic cells, make *CSF1R* a reasonable candidate for further study. Whether these variants change CSF1R signaling, and whether CSF1R inhibition would help a defined group of patients, can only be answered by functional and clinical studies.

### 4.2. ATM Gene

Ataxia telangiectasia mutated gene is located on chromosome 11q22-23 and encodes for protein kinase. This protein involves in cell cycle and DNA damage processes. DNA double-strand breaks (canonical pathway), oxidative stress, topoisomerase cleavage complexes, splicing intermediates, R-loops, and occasionally single-strand DNA breaks all recruit and activate this serine/threonine protein kinase [25]. According to one study, DNA damage from telomere dysfunction activates the ATM protein, which phosphorylates YAP1, leading to increased pro-IL-18 production and triggering inflammatory responses that contribute to inflammatory bowel disease development [26]. MGI databases indicated the association of this gene with colorectal cancer phenotype and GWAS catalog database identified association with lung adenocarcinoma [27] renal carcinoma [28], and prostate carcinoma [29]. Variant effect prediction identified one rare missense variant in *ATM*, p.Phe582Leu, which was predicted to be tolerated by SIFT (0.14, low confidence) and benign by PolyPhen-2 (0). Structural protein modeling illustrated in Figure 6 shows conformational change between the wild and mutant protein types. Both SIFT and PolyPhen-2 predicted this variant to be tolerated, and it is not rare in Middle Eastern populations (Section 3.9), so its contribution to disease is uncertain, and *ATM* is better regarded as a pathway-level candidate than as a gene with a demonstrated damaging variant.

### 4.3. FN1 Gene

*FN1* gene encode for fibronectin 1 protein which involves in immune-cell differentiation. The gene is located on chromosome 2q35. *FN1* is a crucial part of the extracellular matrix, fibronectin comes in a variety of isoforms generated via alternative splicing. *FN1* plays a role in tissue maintenance, embryonic development, and wound healing. Numerous illnesses, such as cancer metastases, and certain inflammatory disorders, have been linked to *FN1* dysregulation [30]. In gastric cancer, FN1 expression affects tumor cell behavior and has prognostic value [31]. *FN1* gene is enriched in ECM-receptor interaction, small cell cancer, immune system, and signaling by interleukin pathways. MGI database indicated an association of this gene with membranoproliferative glomerulonephritis phenotype. GWAS shows association with Coronary Artery disease [32]. WES analysis in our study identified four missense variants in *FN1*: p.Thr2202Ala, p.Asp1450Glu, p.Arg1040Gly and p.Gly313Val. SIFT predicted all of them to be deleterious except p.Thr2202Ala, which was tolerated with low confidence (SIFT 0.14). Structural protein modeling illustrated in Figure 6 shows the conformational changes in the protein structure between mutant and wild type. A previous study reported *FN1* c.938G>T (p.Gly313Val) in patients with early-onset inflammatory bowel disease [33]. The *FN1* p.Gly313Val variant found in our patients is the same amino acid substitution.

### 4.4. IL11 Gene

*IL11* gene encode for interleukin 11 protein which is a cytokine protein involves in immune functions, cell proliferation and signaling pathways. The gene has 5 exons and 4 introns and is located on chromosome 19q13. Enrichment analysis identified the role of this gene in immune system, Cytokine signaling, and signaling by interleukins pathways which indicates the role of this gene in the immune related pathways. In mouse models with conditional expression of interleukin 11 in smooth muscle cells or fibroblasts, severe inflammatory bowel disease with fibrosis was observed, demonstrating that stromal-derived IL11 alone can drive inflammatory bowel disease [34]. WES analysis identified one rare missense variant in *IL11*, p.Met144Thr, predicted to be deleterious by SIFT (0.03) but benign by PolyPhen-2 (0.154). Structural protein modeling shows conformational changes in the protein structure between wild and mutant type of IL11 protein; whether this substitution affects IL-11 function remains to be tested experimentally.

### 4.5. Potential Clinical Relevance

If replicated and validated, these findings could inform clinical practice in several specific ways. As biomarkers, *FN1* and *IL11* encode extracellular and secreted proteins that can be measured in tissue or blood, and *IL11* drives fibro-inflammatory disease in mouse models [34], which makes it a candidate marker of fibrotic or stricturing disease worth testing in CD cohorts. For patient stratification and prediction of treatment response, CSF-1 pathway activation has been reported in CD patients who do not respond to anti-TNF therapy [24], so a CSF1R-related macrophage expression signature, possibly combined with *CSF1R* genotype, could be tested as a predictor of anti-TNF non-response. For precision treatment, CSF1R inhibitors already in clinical development could be evaluated in patients selected by such a signature. Carriers of rare variants in DNA damage response genes such as *ATM* could be followed to see whether they differ in disease course or need closer cancer surveillance, given the role of ATM in genome maintenance [25]. Each of these uses depends on replication of the genetic findings, evidence that the variants change protein function, and prospective validation against clinical outcomes. None is ready for clinical use.

### 4.6. Limitations

This study has several limitations. First, the WES analysis was conducted on a relatively small cohort of 10 IBD patients, which may limit the generalizability of the findings. Second, the study utilized publicly available RNA-seq data rather than matched samples from the same patients who underwent WES. Third, the pathogenic variants identified require functional validation. Finally, the absence of a replication cohort limits confirmation of identified associations.

### 4.7. Future Validation Studies

Several types of study are needed to test the mechanisms proposed here. The variants and gene-level burden should be replicated in a larger IBD cohort with matched population controls, ideally including Saudi and other Middle Eastern patients. Exome or genome sequencing and RNA-seq of inflamed and non-inflamed biopsies should be obtained from the same patients, so that the effect of each variant on expression, including allele-specific expression and expression quantitative trait loci, can be tested directly; such analyses should use mixed models that account for repeated biopsies per patient. The prioritized variants should be studied functionally, for example by CRISPR editing in intestinal epithelial or macrophage cell lines, patient-derived organoids or iPSC-derived macrophages, with readouts of signaling, cytokine release, epithelial barrier function and protein stability. CSF1R inhibition and IL11 blockade could then be tested in colitis models. Prospective clinical studies would be needed to test whether expression or genotype of these genes predicts disease course or response to treatment.

## 5. Conclusions

Our integrative approach, combining WES of 10 IBD patients with transcriptomic data from an independent cohort, nominated *ATM*, *CSF1R*, *FN1*, and *IL11* as candidate genes in IBD. These genes carry rare coding variants in our patients and belong to immune-related pathways such as cytokine and interleukin signaling, and structural modeling predicted conformational changes for several of the variants. The findings are associative and computational. They identify candidate genes, and in the case of *CSF1R* a candidate therapeutic target, that need replication in larger matched cohorts and functional validation before they can inform diagnosis or treatment.

## Figures and Tables

**Table 1:** Total number of variants before and after the filtration in IBD patients. MAF, minor allele frequency; p3_1000G_AF, 1000 Genomes Project phase 3 allele frequency; gnomAD, Genome Aggregation Database.

Patient ID	Total No. of Variants	Effect Filtration	Only Protein Coding	MAF (p3_1000G_AF)	MAF (gnomAD_Exomes_AF)	MAF (gnomAD_Genomes_AF)	Passing Variants	Genes Numbers
1	100,453	39,623	31,564	6753	6301	6268	4847	2711
2	98,511	39,554	31,622	7089	6618	6585	5001	2650
3	97,924	39,174	31,243	6640	6215	6191	4821	2613
4	98,096	39,384	31,361	7008	6538	6507	5051	2609
5	94,674	37,619	29,898	5914	5472	5436	4332	996
6	98,822	39,395	31,385	7644	6761	6725	5163	2869
7	95,660	38,424	30,758	6456	5976	5941	4651	2499
8	96,250	38,065	30,538	6229	5852	5835	4593	2556
9	97,736	38,894	30,705	6342	5936	5905	4751	2496
10	94,856	37,390	30,009	6313	5904	5875	4758	2556
Total of Numbers	972,982	387,522	309,083	66,388	61,573	61,268	47,968	24,555

## Data Availability

The RNA-seq dataset analyzed in this study is publicly available in the NCBI Gene Expression Omnibus (GEO) under accession number GSE193677 (https://www.ncbi.nlm.nih.gov/geo/query/acc.cgi?acc=GSE193677; accessed on 1 August 2025). Due to patient privacy and confidentiality concerns, the whole-exome sequencing data generated in this study are not publicly available. These data can be obtained upon reasonable request directly from the corresponding author, Babajan Banaganapalli (bbabajan@kau.edu.sa). Summary counts of the filtered variants are provided in Table 1.

## Supplementary Materials

The following supporting information can be downloaded at: https://www.mdpi.com/article/10.3390/genes17101306/s1, Table S1. Clinical characteristics of the 10 patients with Crohn’s disease who underwent whole-exome sequencing. Table S2. Sequencing and coverage metrics for the 10 whole-exome samples. Libraries were captured with Agilent SureSelect V7 and sequenced on an Illumina NovaSeq 6000 (2 × 151 bp; run A00718, flow cell HMT2VDSXY). All ten samples were sequenced in the same run and lane. Table S3. Rare missense variants in the four candidate genes identified by whole-exome sequencing of 10 patients with Crohn’s disease. SIFT and PolyPhen-2 predictions are from Ensembl VEP (release 113).
