# Supplementary material for: Integrative Analysis of Whole Exome Sequencing and Gene Expression to Uncover Genetic Mechanisms in Inflammatory Bowel Disease
Source: Genes (Basel). 2026 Oct 20;17(10):1306. doi: 10.3390/genes17101306 (PMC13704380; doi:10.3390/genes17101306)
Supplement: Supplementary file 1 [file genes-17-01306-s001.zip › Supplementary Table S1.pdf]

Table S1. Clinical characteristics of the 10 patients with Crohn's disease who underwent whole-exome sequencing.

**Patient**	**Sex**	**Age at diagnosis**	**Key clinical features**	**Treatment**
P1	M	22 y	Esophageal erosions, hemorrhagic gastropathy	5-ASA, AZA, IFX
P2	M	17 y	Ileal stricture, enterocolonic fistula, pseudopolyps; prior tuberculosis	—
P3	M	25 y	—	Adalimumab
P4	F	~28 y	Colectomy, ostomy	—
P5	M	25 y	—	IFX
P6	M	~29 y	Bloody diarrhea, vomiting	IFX
P7	M	~23 y	Colectomy at 26 y	—
P8	F	6 mo	Pan-ileocolonic ulceration, osteopenia, eczema, hyperlipidemia	5-ASA, AZA, IFX
P9	F	6 mo	Severe esophageal stenosis, pustular skin lesions, FTT	5-ASA, AZA
P10	F	36 mo	Moderate to severe colitis, FTT	5-ASA

5-ASA, mesalamine; AZA, azathioprine; IFX, infliximab; FTT, failure to thrive; —, not recorded.
