# Supplementary material for: Integrative Analysis of Whole Exome Sequencing and Gene Expression to Uncover Genetic Mechanisms in Inflammatory Bowel Disease
Source: Genes (Basel). 2026 Oct 20;17(10):1306. doi: 10.3390/genes17101306 (PMC13704380; doi:10.3390/genes17101306)
Supplement: Supplementary file 1 [file genes-17-01306-s001.zip › Supplementary Table S3.pdf]

Table S3. Rare missense variants in the four candidate genes identified by whole-exome sequencing of 10 patients with Crohn's disease. SIFT and PolyPhen-2 predictions are from Ensembl VEP (release 113).

Gene	Transcript	HGVS c.	Protein change	GRCh38 position	rsID	SIFT (score)	PolyPhen-2 (score)	gnomAD AF, all	gnomAD AF, Middle Eastern	Genotype	Carriers (n of 10)
CSF1R	NM_001288705.3	c.2788A>T	p.Ser930Cys	chr5:150054200	rs1391201688	Deleterious (0.02)	Possibly damaging (0.544)	2.1×10^{-6}	0	Homozygous	2
CSF1R	NM_001288705.3	c.368C>G	p.Ala123Gly	chr5:150080276	rs140924076	Deleterious (0)	Benign (0.289)	3.1×10^{-4}	4.7×10^{-3}	Homozygous	3
ATM	NM_000051.4	c.1744T>C	p.Phe582Leu	chr11:108251973	rs2235006	Tolerated, low confidence (0.14)	Benign (0)	8.0×10^{-4}	2.3×10^{-2}	Homozygous	1
FN1	NM_212482.4	c.6604A>G	p.Thr2202Ala	chr2:215372019	rs755094788	Tolerated, low confidence (0.14)	—	3.4×10^{-6}	0	Homozygous	1
FN1	NM_212482.4	c.4350T>A	p.Asp1450Glu	chr2:215386951	Not in dbSNP	Deleterious (0)	—	Absent	Absent	Homozygous	1
FN1	NM_212482.4	c.3118A>G	p.Arg1040Gly	chr2:215404524	rs547314154	Deleterious (0.01)	—	6.4×10^{-4}	3.5×10^{-4}	Homozygous	1
FN1	NM_212482.4	c.938G>T	p.Gly313Val	chr2:215425192	rs1431183911	Deleterious (0)	—	3.4×10^{-6}	0	Homozygous	2
IL11	NM_000641.4	c.431T>C	p.Met144Thr	chr19:55366176	rs552736613	Deleterious (0.03)	Benign (0.154)	3.8×10^{-5}	5.3×10^{-4}	Homozygous	1

Transcripts are the MANE Select transcripts used for annotation; positions are on GRCh38. Allele frequencies (AF) are from gnomAD v4.1 exomes, retrieved through the Ensembl VEP. Variant filtering used gnomAD v2.1; v4.1 is shown here because v2.1 has no Middle Eastern population.
