# Supplementary material for: Integrative Analysis of Whole Exome Sequencing and Gene Expression to Uncover Genetic Mechanisms in Inflammatory Bowel Disease
Source: Genes (Basel). 2026 Oct 20;17(10):1306. doi: 10.3390/genes17101306 (PMC13704380; doi:10.3390/genes17101306)
Supplement: Supplementary file 1 [file genes-17-01306-s001.zip › Supplementary Table S2.pdf]

Table S2. Sequencing and coverage metrics for the 10 whole-exome samples. Libraries were captured with Agilent SureSelect V7 and sequenced on an Illumina NovaSeq 6000 (2 × 151 bp; run A00718, flow cell HMT2VDSXY). All ten samples were sequenced in the same run and lane.

**Sample**	**Total reads (M)**	**Mean read length (bp)**	**Q30 (%)**	**Duplicate-removed reads retained (%)**	**On-target reads (%)**	**Mean on-target depth (×)**	**≥10× (%)**	**≥20× (%)**	**≥30× (%)**
Sample 1	73.62	148.62	93.3	91.5	64.2	102.1	98.0	94.4	88.5
Sample 2	71.40	148.76	93.8	89.1	67.2	101.3	96.7	90.6	82.2
Sample 3	56.33	148.8	93.7	91.9	67.4	82.5	97.5	92.4	84.6
Sample 4	57.76	147.97	93.7	91.3	63.7	79.1	96.5	89.9	80.9
Sample 5	58.01	148.75	94.0	92.5	72.2	92.5	96.8	91.4	84.2
Sample 6	77.98	149.42	93.8	91.6	67.6	114.4	98.4	95.5	91.2
Sample 7	62.32	148.89	94.0	92.0	71.0	96.7	96.7	90.9	83.5
Sample 8	65.93	148.38	94.0	90.9	69.1	98.2	96.8	91.9	85.1
Sample 9	59.43	148.84	93.5	90.5	56.6	71.9	96.6	88.5	77.2
Sample 10	53.04	146.67	93.6	91.6	67.4	75.4	95.5	86.3	75.1
**Mean (10 samples)**	63.6	148.5	93.7	91.3	66.6	91.4	97.0	91.2	83.2
